# Thermodynamic Calculation among Cerium, Oxygen, and Sulfur in Liquid Iron

**DOI:** 10.1038/srep35843

**Published:** 2016-10-21

**Authors:** Fei Pan, Jian Zhang, Hao-Long Chen, Yen-Hsun Su, Yen-Hao Su, Weng-Sing Hwang

**Affiliations:** 1 Department of Materials Science and Engineering, National Cheng Kung University, Tainan 70101, Taiwan; 2 Physics Department, Technische Universität München, Munich 85748, Germany; 3 Physics Department, Ludwig-Maximilians-Universität München, Munich 80799, Germany; 4 Chengdu Base, Panzhihua Iron & Steel Research Institute Co., LTD, Chengdu 610399, China; 5 Department of Electronic Engineering, Kao Yuan University, Kaohsiung 82151, Taiwan; 6 Steelmaking Process Development Section, China Steel Corporation, Kaohsiung 81233, Taiwan

## Abstract

Thermodynamic calculation has been applied to predict the inclusion formation in molten SS400 steel. When the Cerium addition in liquid iron is 70 ppm and the initial Oxygen and Sulphur are both 110 ppm, the formation of oxides containing Cerium would experience the transformation from Ce_2_O_3_ to CeO_2_ and also the formation of sulfides containing Cerium would experience the transformation from CeS to Ce_2_S_3_ and then to Ce_3_S_4_. Below 2000 K the most thermodynamic stable matter is CeO_2_ and the less thermodynamic stable inclusion is CeS. Only when the amount of [O] is extremely low and the amount of [S] and [Ce] is relatively high, Ce_2_S_3_ has the possibility to form.

Rare earth (RE) metals have many applications^1^,^2^,^3^,^4^,^5^ and their addition to molten iron has attracted increasing research attention^6^. Such addition affects inclusion structures^7^ and can be used to purify steel^8^. The conjugation between oxygen and RE metals^9^ and between sulfur and RE metals^10^ is very strong. A lot of research^11^,^12^,^13^ has been done on the equilibrium relation between O, S, and RE metals. It has been found that extremely low oxygen and sulfur concentrations in steel can be achieved via the addition of an RE metal^14^. A lot of research^15^,^16^,^17^,^18^ has also been done on steel deoxidization and desulfurization via titanium and magnesium. RE metals can be used to deoxidize and desulfurize steel to control inclusion size and chemical composition. Few studies have performed thermodynamic calculations on the use of cerium to modify inclusions.

This paper focuses on the thermodynamic calculations of the cerium-oxygen-sulfur system in molten SS400 steel. The formation conditions of CeS, Ce_2_S_3_, Ce_3_S_4_, CeO_2_, and Ce_2_O_3_ in molten steel are examined using Wagner’s relation and Lupis’s relation based on the Gibbs free energy change. The transformation mechanism is analyzed by determining the thermodynamic conditions of Ce-desulfurized and Ce-deoxidized steel. The segregation of Ce_2_O_3_ in molten iron is also analyzed. In addition, a model for predicting the formation of various inclusions is established for SS400 steel with cerium addition.

## Calculations

The thermodynamic calculations of the Ce-O-S system are based on Wagner’s relation^19^ and Lupis’ relation^20^. These calculations were implemented in C++. The segregation of Ce_2_O_3_ in molten SS400 steel, whose chemical composition is shown in Table 1, was calculated in Matlab 2015a.

The Ce-O-S system is the thermodynamic relation between the dissolved Oxygen, Sulphur and Cerium in liquid iron to explore the formations of inclusions containing Cerium. The first stage for thermodynamic calculation is to derive the thermodynamic equations for the inclusion formations by Wagner’s relation^19^ and Lupis’ relation^20^. Then the second stage is to use C++ programming software to derive the unknown chemical composition values for every equation.

## Results and Discussion

For the addition of cerium into molten SS400 steel, the reactions of [O], [S], and [Ce] are of interest because Ce has strong affinity with S and O. As reported previously^21^, when *w*(RE)/(*w*[O] + *w*[S]) = 3.9, the function of cerium is optimal. To determine the separation sequence for various oxides and sulfides of cerium, the amount of cerium in the calculations was set as 1 mol to compare the Gibbs free energy of formation for various inclusions, which can be derived as:


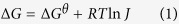



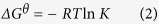


where *J* denotes the reaction quotient (unitless), ∆*G* is the Gibbs free energy change of reaction (J/mol), ∆*G*^*θ*^ denotes the Gibbs free energy change of reaction for unmixed reactants and products at standard conditions (J/mol), *R* is the gas constant (J·mol^−1^·K^−1^), *T* is temperature (K), and *K* is the equilibrium constant (unitless).

The Gibbs free energy of oxides, sulfides and oxysulfides of cerium are shown in Table 2 14,21,22,23,24,25,26.

**Figure 1:**
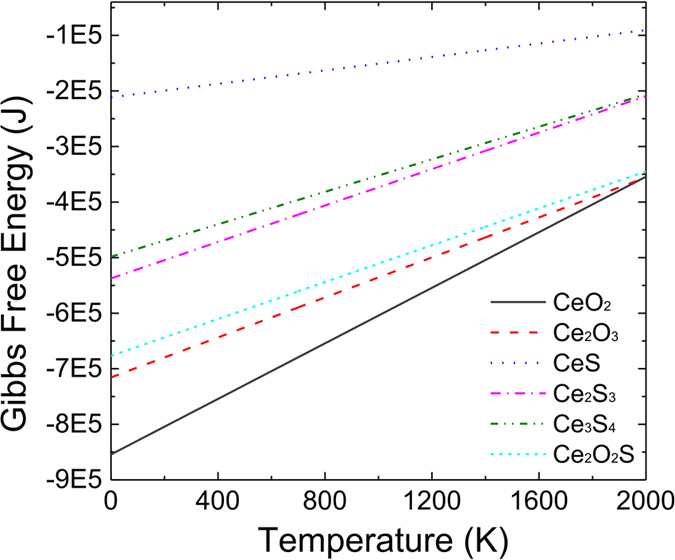
Gibbs free energy of formation for various oxides and sulfides containing cerium at various temperatures.

Below 2000 K, the most thermodynamically stable inclusion was CeO_2_, as shown in Fig. 1. Therefore, CeO_2_ likely formed in the molten iron when the temperature reached the simulated steelmaking temperature of 1873 K. In Fig. 1, it could be read that the least thermodynamic stable inclusion is CeS and the thermodynamic stale sequence of the possible inclusion formed in liquid steel is CeO_2_ > Ce_2_O_3_ > Ce_2_O_2_S > Ce_2_S_3_ > Ce_3_S_4_ > CeS. However, the most thermodynamically stable matter does not guarantee the formation of CeO_2_, because the formation of oxides containing cerium are controlled not only by the equilibrium constant but also by the concentrations of cerium and oxygen in the molten iron. That is to say, the formation of CeO_2_ at 1873 K is also determined by the solubility product of CeO_2_ and the concentration of cerium and oxygen, even though the Gibbs Free Energy of CeO_2_ is the lowest at 1873 K.

The activities and activity coefficient of Ce, O and S can be written as Eqs (9) and (10) from Wagner’s relation^7^ and Lupis’ relation^20^ as follow,


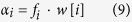



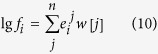


where *f*_*i*_ is the Henrian activity coefficient of component *i* relative to the dilute solution and 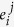 is the first-order interaction parameter of *i* on *j* in molten iron; *w*[*i*] and *w*[* j*] are the mass percentages of elements *i* and *j*, respectively (Table 3); *α*_*i i*_s the activity of element *i*.

**Figure 2:**
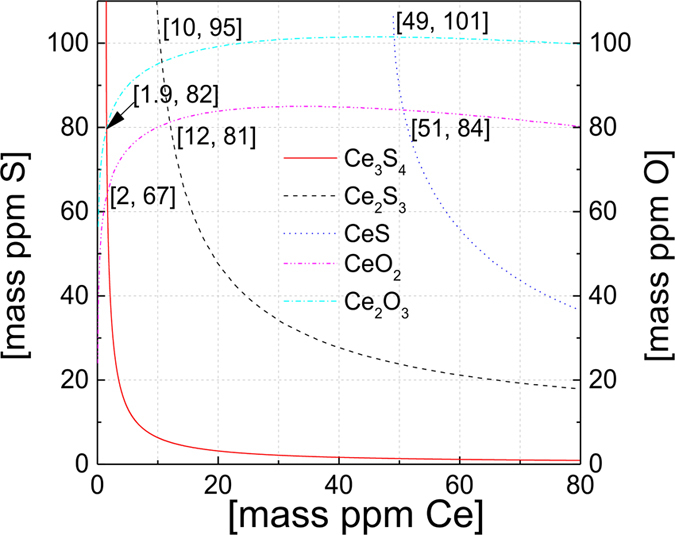
Deoxidation and Desulphurization with Cerium in liquid iron at 1873 K.

By using data^22^,^23^ from Tables 2 and 3, the following curves for Ce-S and Ce-O in Fig. 2 can be calculated. The key to derive every line in Fig. 2 is the relation of equilibrium constant, Gibbs free energy and the amount of the chemical compositions for every possible inclusion according to Wagner’s relation^19^ and Lupis’ relation^20^. When the equilibrium constant is linked to the amount of the chemical compositions for every possible inclusion, equations for Fig. 2 can be obtained. When the weight percentage of cerium, oxygen and sulphur are known in the molten iron at 1873 K, the main inclusion formed would be found in Fig. 2. As shown in Fig. 2, if the cerium addition in liquid iron is 70 ppm and the initial oxygen and sulphur are both 110 ppm, the formation of oxides containing cerium would experience the transformation from Ce_2_O_3_ to CeO_2_ and also the formation of sulfides containing cerium would experience the transformation from CeS to Ce_2_S_3_ and then to Ce_3_S_4_. From Fig. 2, when the temperature of molten iron reached 1873 K, Ce_3_S_4_ is the main product, as the amount of cerium in molten iron is high and the amount of sulphur is relatively lower compared to the formation of CeS and Ce_2_S_3_.

In order to investigate the formation conditions of Ce_2_O_3_, Ce_2_S_3_ and Ce_2_O_2_S, the doubly saturated curve with Ce_2_O_3_/Ce_2_O_2_S and Ce_2_S_3_/Ce_2_O_2_S are calculated, using the thermodynamic data derived in Tables 2 and Equation 1, 2.

**Figure 3:**
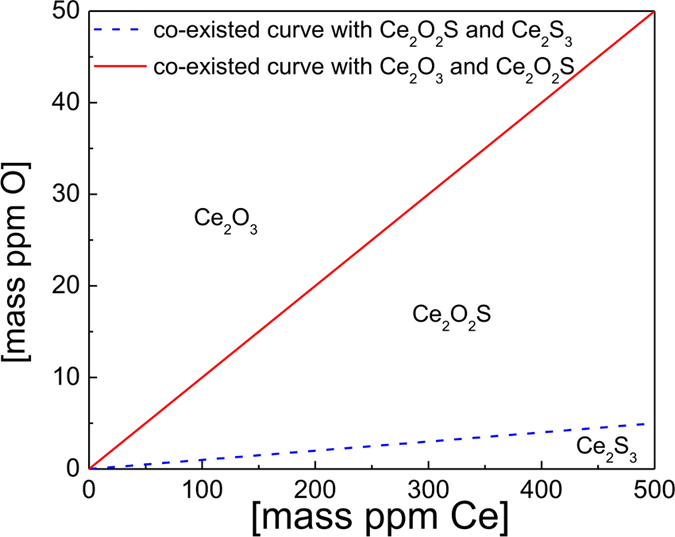
Relationship of [O] and [S] when Ce_2_O_2_S, Ce_2_O_3_, and Ce_2_S_3_ can form as stable compounds in molten iron at 1873 K.

In molten iron, it is assumed that 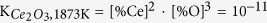 and 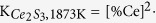 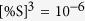. Based on the reaction Ce_2_O_2_S + [O] = Ce_2_O_3_ + [S], it is found that [%S] = 10[%O] when Ce_2_O_2_S and Ce_2_O_3_ coexist. When Ce_2_O_2_S and Ce_2_S_3_ coexist in molten iron, it is derived that [%S] = 100[%O], based on the thermodynamic calculation of the reaction Ce_2_S_3_ + 2[O] = Ce_2_O_2_S + 2[S]. Figure 3 was derived from the above calculations. In Fig. 3, it can be concluded that Ce_2_O_3_ and Ce_2_O_2_S can exist in molten iron in a wide amount range of [Ce], [O] and [S]. More importantly, only when the amount of [O] is extremely low and the amount of [S] and [Ce] is relatively high, Ce_2_S_3_ has the possibility to form.

Cerium is a perfect deoxidizer and desulfurizer for steel purification. Compared with other elements, for example Aluminum, Titanium, Magnesium and Calcium^27^,^28^, which can also deoxidize and desulfurize, cerium can formed a complex compound Ce_2_O_2_S which contains Oxygen and Sulphur together. The formation possibility of Ce_2_O_2_S has been verified by Hu’s research^29^ when they studied the effect of Ce addition on the C-Mn steel microstructure. It is reproted by Wang^26^ that Ce_2_O_3_ is easier to form in molten iron when the iron molten temperature is 1873 K. However, the thermodynamic conditions were changed when the temperature decreases from 1873 K to solidus temperature. On the other hand, when the temperature of molten iron decreases to that at which solid steel starts to form, the cerium and oxygen in the molten iron begin to segregate. Their amounts are respectively:


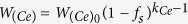



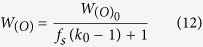


where *W*_(*Ce*)_ and *W*_(*O*)_ are the percentage amounts of cerium and oxygen of molten iron during the molten iron solidification, respectively; 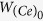
and 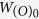 are the initial percentage amounts of cerium and oxygen in the liquid phase, respectively; *k*_*Ce*_ (=0.005) and *k*_*O*_ (=0.022) are the solvent partition coefficients at equilibrium for cerium and oxygen, respectively; *f*_*s*_ is the solid fraction.

The solidus temperature of SS400 is 1777 K. The solubility product of the Ce_2_O_3_ formed in molten iron can be expressed as:


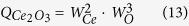


The solubility product of the Ce_2_O_3_ formed in molten iron at equilibrium can be expressed as:


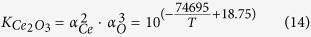


**Figure 4:**
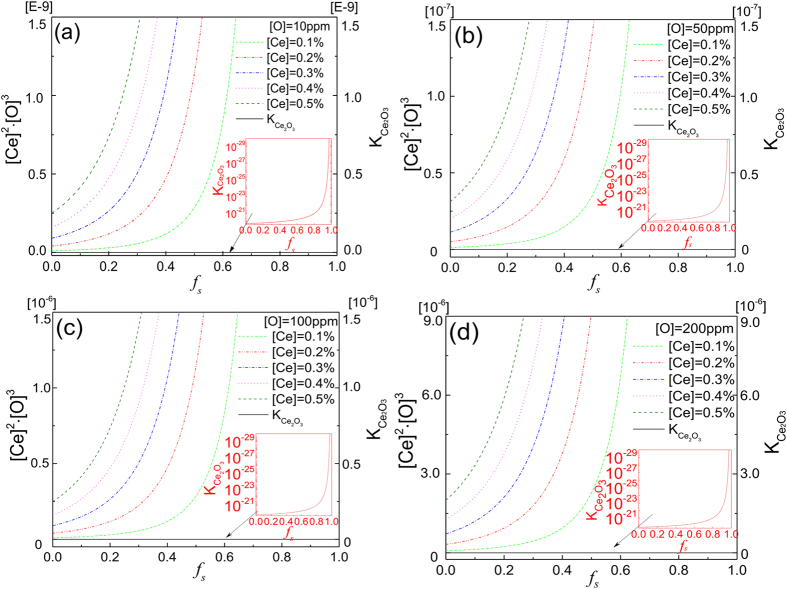
Segregation of Ce_2_O_3_ in solid and liquid phases when α_[*o*]_ is (**a**) 10, (**b**) 50, (**c**) 100, and (**d**) 200 ppm.

From Eqs (11) to (14), the solubility products versus solidification ratio (f_s_) are plotted in Fig. 4. In Fig. 4(a), where the simulated oxygen concentration in liquid steel is 10 ppm and the cerium concentration varies from 0.1% to 0.5%, the solubility products versus solidification ratio (f_s_) are plotted with the varying cerium concentration (shown in the colorful lines of Fig. 4(a)) and the equilibrium constant of Ce_2_O_3_ (K_Ce2O3_) versus solidification ratio f_s_ is curved as the black solid line in Fig. 4(a). It is read in Fig. 4(a) that the colorful lines are all in the above of the black solid line, which means Ce_2_O_3_ prefers to segregate in liquid phase with the 10 ppm Oxygen concentration in liquid iron. Moreover, the same conclusion can be drawn from the similar Fig. 4(b–d) with 50 ppm, 100 pmm, 200 ppm oxygen concentration in liquid iron. The inset red diagrams in Fig. 4(a–d) are the detailed solid black curves appeared in Fig. 4(a–d). Figure 4 shows that when the oxygen concentration in molten iron was increased from 10 to 200 ppm and the cerium concentration was in the range of 0.1% to 0.5%, Ce_2_O_3_ preferred to segregate in the liquid phase.

## Conclusion

By the addition of cerium in molten SS400 steel, when the temperature of molten iron reached 1873 K, at the same time that the Cerium addition in liquid iron is 70 ppm and the initial Oxygen and Sulphur are both 110 ppm, the formation of oxides containing Cerium would experience the transformation from Ce_2_O_3_ to CeO_2_ and also the formation of sulfides containing Cerium would experience the transformation from CeS to Ce_2_S_3_ and then to Ce_3_S_4_. Below 2000 K the most thermodynamic stable matter CeO_2_ and the least thermodynamic stable inclusion is CeS. And the thermodynamic stable sequence of the possible inclusions formed in liquid steel is CeO_2_ > Ce_2_O_3_ > Ce_2_O_2_S > Ce_2_S_3_ > Ce_3_S_4_ > CeS. Only when the amount of [O] is extremely low and the amount of [S] and [Ce] is relatively high, Ce_2_S_3_ has the possibility to form. With the amount of oxygen in molten iron increasing from 10 ppm to 200 ppm and the amount range of cerium increasing from 0.1% to 0.5%, Ce_2_O_3_ prefers to segregate in liquid phase all the time.

## Additional Information

**How to cite this article**: Pan, F. *et al.* Thermodynamic Calculation among Cerium, Oxygen, and Sulfur in Liquid Iron. *Sci. Rep.* **6**, 35843; doi: 10.1038/srep35843 (2016).

## Figures and Tables

**Table 1:** Chemical composition of SS400 steel (wt. %).

C	Si	Mn	P	S	O
0.14	0.26	0.90	0.02	0.03	0.018

**Table 2:** Formation equations and Gibbs free energy of oxides, sulfides and oxysulfides of cerium^14^,^21^,^22^,^23^,^24^,^25^,^26^.

Reaction	Standard Gibbs Free energy, J/mol	No.
[Ce] + 2[O] = CeO_2_(s)	ΔG^θ^ = −854270 + 250T	(3)
[Ce] + 3/2[O] = 1/2Ce_2_O_3_(s)	ΔG^θ^ = −715560 + 180T	(4)
[Ce] + [S] = CeS(s)	ΔG^θ^ = −211390 + 60.5T	(5)
[Ce] + 3/2[S] = 1/2Ce_2_S_3_(s)	ΔG^θ^ = −537290 + 164T	(6)
[Ce] + 4/3[S] = 1/3Ce_3_S_4_(s)	ΔG^θ^ = −498480 + 146.3T	(7)
[Ce] + [O] + 1/2[S] = 1/2Ce_2_O_2_S(s)	ΔG^θ^ = −676795 + 166T^18^	(8)

**Table 3:** First-order interaction parameter 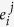 of cerium, oxygen, and sulfur at 1873 K^30^.

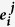	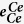	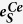	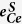	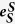	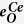	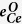	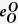
T = 1873 K	0.0039	−9.1	−40	−0.046	−64	−560	−0.17
